# Prolonged Induction of miR-212/132 and REST Expression in Rat Striatum Following Cocaine Self-Administration

**DOI:** 10.1007/s12035-016-9817-2

**Published:** 2016-03-05

**Authors:** Anna Sadakierska-Chudy, Małgorzata Frankowska, Joanna Miszkiel, Karolina Wydra, Joanna Jastrzębska, Małgorzata Filip

**Affiliations:** Laboratory of Drug Addiction Pharmacology, Institute of Pharmacology Polish Academy of Sciences, Krakow, Poland

**Keywords:** Addiction, Cocaine self-administration, Extinction training, Gene expression, miRNAs, Striatum, Synaptic proteins

## Abstract

Chronic exposure to cocaine in vivo induces long-term synaptic plasticity associated with the brain’s circuitry that underlies development of repetitive and automatic behaviors called habits. In fact, prolonged drug consumption results in aberrant expression of protein-coding genes and small regulatory RNAs, including miRNAs that are involved in synaptic plasticity and neuroadaptations. However, the mechanisms mediating cocaine use disorder are still not fully understood. The present study is designed to examine the expression of *miR-124*, *miR-132*, *miR-134*, and *miR-212*, as well as the levels of the Ago2, Pum2, and REST mRNAs and proteins implicated in their regulation. We applied rat cocaine self-administration (SA) and extinction training procedures with a yoked triad to assess the changes in the levels of four miRNAs and three protein-coding genes and corresponding proteins in the dorsal striatum. We demonstrated that elevated expression of mature *miR-212* and *miR*-*132* is long-lasting and persists in the drug-free period (till 10-day abstinence). Moreover, mRNA and protein of REST, a regulator of neuronal transcription, was raised selectively in cocaine self-administering rats and *Ago2* transcript decreased after cocaine treatment. Unexpectedly, the expression level of Ago2 and Pum2 proteins changed only in the active cocaine-receiving animals. These results point out the important aspects of long-lasting alterations in microRNAs, genes, and protein expressions involved in the control of synaptic plasticity associated with reward and motivation learning related to cocaine addiction.

## Introduction

Substance use disorder (drug addiction) is defined as a chronic, relapsing brain disease that is characterized by uncontrolled drug consumption and seeking, despite its harmful consequences. Due to the complex nature of drug addiction, our understanding of the mechanisms mediating addictive behavior is still incomplete. Currently, addiction to cocaine and other drugs of abuse is considered to be a drug-induced disorder of neuroplasticity [1]. In fact, prolonged drug consumption results in aberrant expression patterns for the genes that encode both proteins and microRNAs (miRNAs) that are involved in synaptic plasticity and neuroadaptations [2, 3]. In addition, several miRNAs may alter the dendritic spine morphology [4], initiate long-term potentiation (LTP) [5] and drive addictive behaviors [6].

miRNAs are single-stranded, short (∼22 nt), non-coding RNA molecules that regulate gene expression at the posttranscriptional level [7, 8]. It is well-known that a single miRNA may target several mRNAs [9]; conversely, a single mRNA may be targeted by different miRNAs [10]. The capacity of the miRNAs to target many transcripts suggests that they have an important role in the complex regulatory network that fine tunes gene expression [11]. The brain-specific miRNAs appear to be differentially distributed across the regions as well as cell types and synaptic compartments [11, 12].

Recently, it was demonstrated that four miRNAs (*miR-124*, *miR-132*, *miR-134*, and *miR-212*) are especially important for neuronal function, plasticity, and/or substance use disorder. Thus, *miR-124* (also known as *miR-124a*) is an exclusively expressed neuronal miRNA and its downregulation through cAMP response element-binding protein (CREB)-dependent transcription may contribute to constrain long-term plasticity. Furthermore, *miR-124* is predicted to target of REST complex that itself acts as a negative regulator of *miR-124* via RE1 sequences [13]. A recent study showed that chronic passive cocaine treatment downregulates the brain-specific *miR-124* transcript in rat striatum and nucleus accumbens (NAc) [4]. Another brain-specific miRNA is *miR-134* that overexpression disturbs synaptic growth accompanying synaptic plasticity [8]. As uncovered by Fiore et al., *miR-134* may regulate protein synthesis by regulating RNA binding protein Pumilio-2 (Pum2) [14], while other studies have suggested that *miR-134* interacts with *PUM2* buffering PUM2 protein levels that may be crucial for maintenance of LTP [5, 15].

Finally, *miR-212/132* cluster, composed of two miRNAs sharing close sequences, was identified as a target of CREB and REST protein that control its own expression [16]. The in vivo studies demonstrated that *miR-132* expression is regulated by neuronal activity and that the miRNA influencing structural and synaptic plasticity may regulate experience-dependent plasticity [17]. Recent preclinical observations reported that either acute cocaine treatment [18] or extended access to cocaine self-administration [19, 20] significantly increases *miR-132* expression in rodent striatal neurons. Similarly, the level of *miR-212* is relatively high in the rat striatum [21], where extended access to cocaine self-administration increases its expression level. Importantly, it was established that knockdown of *miR-212* enhances cocaine rewarding properties in self-administration procedures [19].

Importantly, miRNA biogenesis and function are regulated by multiple level-complex enzymatic machinery, engaged in miRNA processing and in drug abuse and addiction. Thus, argonaut-2 (Ago2), a member of the Argonaute family of proteins, is the catalytic component of the RISC complex that mediates miRNA processing and miRNA-induced target mRNA silencing. Knockout of *Ago2* in D2-expressing accumbal neurons significantly attenuates the motivational aspect of cocaine self-administration [22], while chronic cocaine exposure elevates the level of its mRNA and protein in rat striatum [23].

Another miRNA regulator and an RNA-binding protein, *Pum2*, controls RNA dendritic granules, regulates mRNA transport, stability, or translation, and is implicated in the regulation of neuronal homeostasis and plasticity [14, 24]. Recent studies revealed that *Pum2* mRNA is a direct target of *miR-134* [14] and metabolic stress, which recruits Pum2 into stress granules (SGs) [25]. Interestingly, both the Pum2 mRNA and protein are localized to dendrites, where the protein acts as a translational repressor [25] and as a modulator of activity-dependent dendritogenesis [14].

The expression of target genes and ncRNAs (e.g., miRNAs and lncRNAs) is under the control of the transcriptional and epigenetic regulatory factor REST (RE1 silencing transcription factor) [26]. Interestingly, REST may influence its own expression via complex feedback mechanisms that are regulated by miRNAs (i.e., a double-negative feedback loop) [27]. As suggested, high REST levels may be detrimental for neuronal development, since its overexpression significantly reduces dendritic length and arborization in primary mouse neurons [28]. Chronic cocaine administration significantly increased *REST* mRNA level in rat striatum and NAc [4].

Consistent with the above findings, in this study, we analyzed the expression of *miR-124*, *miR-132*, *miR-134*, and *miR-212*, as well as the levels of the Ago2, Pum2, and REST mRNAs and proteins following cocaine self-administration (SA) and its withdrawal with using extinction training (neither cocaine delivery nor the presentation of the conditioned stimulus) in rats. Extinction training is considered as a new form of learning that facilitates adjustment behavior to a changing environment [29, 30]. Like other forms of learning, both cocaine self-administration and extinction training activate the gene expression, neurochemical, neurophysiological, and structural changes that are essential for memory consolidation and/or retrieval. An understanding of the genetic and molecular underpinnings of different stages of cocaine use disorder is the key to the future development of pharmacological treatments for cocaine abuse.

## Materials and Methods

### Behavioral Experiments

#### Animals

**Fig. 1:**
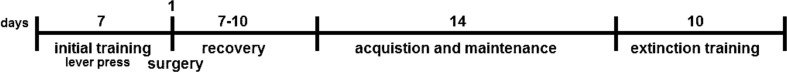
Schematic of the procedure for the behavioral studies

Male Wistar rats (280–300 g; Charles River, Germany) were housed in groups of five per cage during the initial training or individually after surgery and during SA/extinction training. The animals were housed under standard laboratory conditions (temperature 20 ± 1 °C; relative humidity 45 ± 5 %; 12-h light/dark cycle, lights on at 6:00 a.m.). Food and water was available ad libitum, unless indicated otherwise. All experimental procedures took place during the light cycle between the hours of 8:00 a.m. and 3:00 p.m. The steps of all procedures are shown in Fig. 1. All of the methods complied with the European Directive 2010/63/EU and were approved by the Bioethical Committee at the Institute of Pharmacology, Polish Academy of Sciences, Krakow.

#### Drugs

Cocaine hydrochloride (Sigma-Aldrich, USA) was dissolved in sterile 0.9 % NaCl and administered intravenously (i.v.).

#### Initial Training

The day before the initial training, the animals were water deprived for 18 h and then were trained for 1 week with increasing fix ratio (FR) requirements (FR1, FR3, and finally, FR5). The amount of water each rat received was restricted to that given during the daily training sessions. After this lever press training, water deprivation ended and animals had free access to water throughout the subsequent tests.

#### Surgery

The next day following lever-press training animals were anesthetized with ketamine HCl (75 mg/kg, i.p.; Bioketan, Biowet, Poland) and xylazine HCl (5 mg/kg, i.p.; Sedazin, Biowet, Poland), and a silastic catheter was inserted into the right jugular vein and passed subcutaneously onto the rat’s back, posterior to the shoulder blades, as previously described [31]. After surgery, the catheters were flushed once daily with 0.1 ml of an antibiotic solution of cefazolin (10 mg/kg; Tarfazolin, Polfa, Poland) dissolved in heparinized saline (70 U/ml; Polfa, Poland) or with heparinized saline. The animals were allowed to recover from surgery for 7 days prior to the self-administration sessions.

#### Self-Administration and Extinction Training

After recovery from surgery, the intravenous SA procedures were conducted in standard operant chambers (MedAssociates, St. Albans, USA). Each test chamber was equipped with two levers: an active lever, which when pressed, resulted in drug delivery, and an inactive lever, which when pressed, had no consequences. The experimental events were scheduled and data collection was controlled by the MedPC IV software (MED Associates Inc., Vermont, USA).

**Fig. 2:**
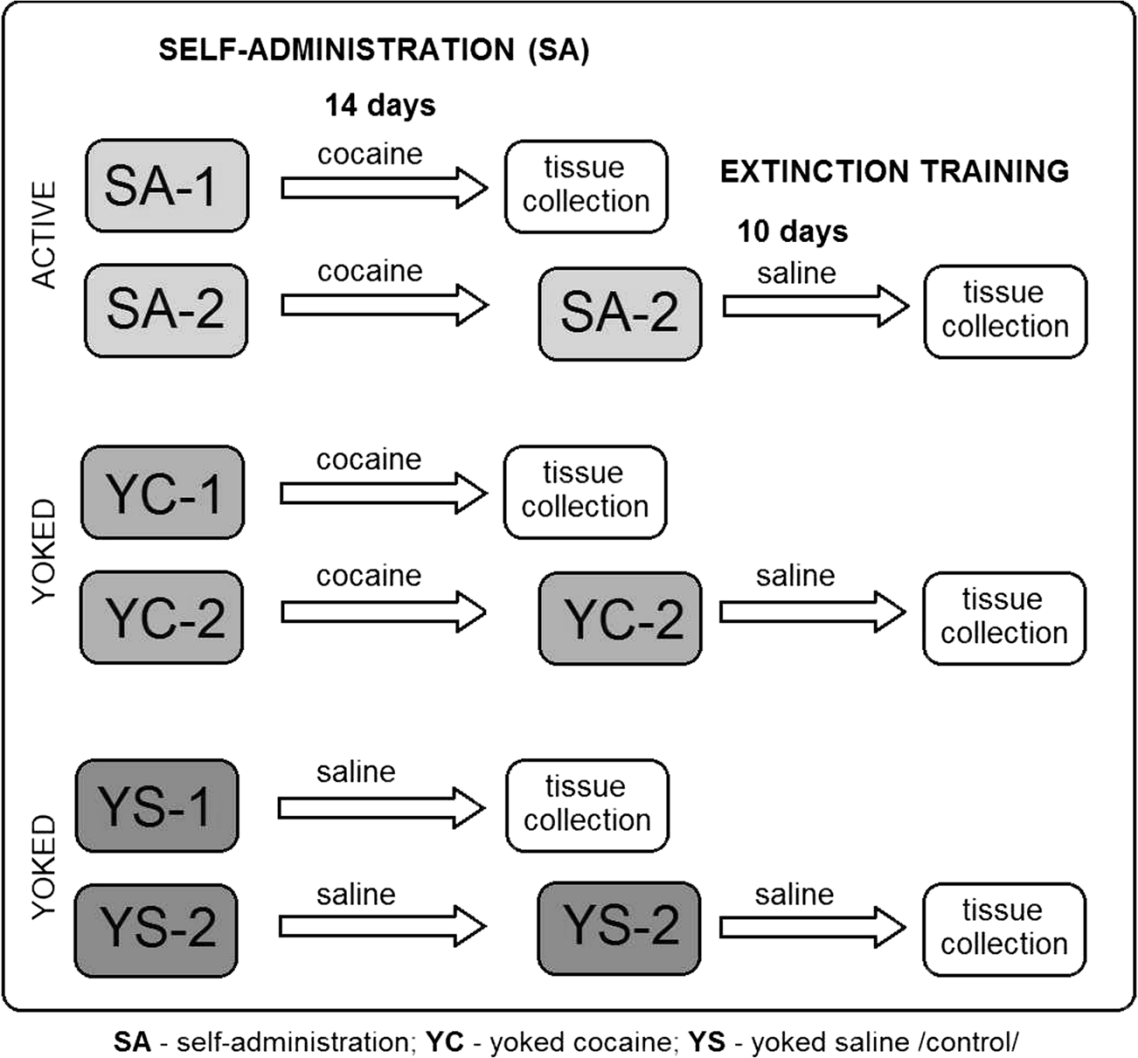
A schematic of the two experimental phases

The SA training was performed during 2-h daily sessions on an FR5 schedule of reinforcement. Presses on the active lever resulted in a 5-s infusion of 0.1 ml of cocaine (0.5 mg/kg/infusion) and a 5-s presentation of the conditioning stimuli directly above the active lever (white light and tone (2000 Hz; 15 dB above the ambient noise levels)). Each infusion was followed by a 20-s time-out period, during which time the animals’ active lever presses were recorded and included in the analysis, but had no programmed consequences. All SA sessions were performed for 14 days. The criterion for acquisition was a 3-day period during maintenance in which the number of active lever presses varied by 10 % or less. Rats that acquired cocaine SA (SA-1) and their yoked cocaine (YC-1) and yoked saline (YS-1) controls were sacrificed immediately after the last 2-h SA session, whereas the remaining groups were subjected to extinction training (for details, see Fig. 2). As shown in Fig. 2, the animals that self-administered cocaine (SA-2) and those that received yoked cocaine (YC-2) and yoked saline (YS-2) underwent 10-day extinction training. The extinction training sessions occurred in the same operant chambers and lasted for 2 h daily, with no cocaine delivery (saline was substituted for cocaine) and no presentation of the conditioning stimuli. On the 10th day of the extinction phase, the animals were sacrificed immediately following the last 2-h extinction session.

The dissected rat brain structures were rapidly placed on dry ice and frozen at −80 °C for further analyses.

#### Yoked Self-Administration Procedure

The use of the yoked procedure allowed us to distinguish the pharmacological effects of cocaine from those related to motivation and the cognitive processes evoked by active drug intake. Three rats were tested simultaneously in different conditions: one rat actively self-administered cocaine and the other two rats were passively exposed to an infusion of either cocaine (yoked cocaine) or saline (yoked saline). The yoked cocaine rats received a drug infusion at the same dose and rate as the self-administration group. The levers pressed by the yoked rats were recorded but had no programmed consequences.

### Molecular Experiments

#### RNA Isolation

The total RNA was extracted from the frozen dorsal striatum (10–15 mg) using TRIzol reagent (Life Technologies, USA) according to the manufacturer’s instruction. Briefly, the brain samples were homogenized using the TissueLyser II (Qiagen, USA) (2 × 2 min at 20 Hz) in the presence of a stainless steel bead and 200 μl of TRIzol. The RNA was precipitated by adding an equal volume of isopropanol to the aqueous phase, and the pellet was washed twice with 1 ml of cold 75 % ethanol. The air-dried pellet was resuspended in 20 μl of RNase-free water. To remove the trace genomic DNA contamination, the total RNA was treated with Deoxyribonuclease I (DNase I) Amplification Grade (Sigma, USA) according to the manufacturer’s protocol. To assess the quality and quantity of the RNA, the samples were analyzed using gel electrophoresis and spectrophotometry (NanoDrop ND-1000, Thermo Scientific, USA) and then stored at −80 °C until further use.

#### RT-qPCR for miRNA Expression

The total RNA (20 ng) and miRNA-specific stem-loop RT primers (Applied Biosystems, USA) were used for the reverse transcription reactions. The cDNAs were then synthesized with the TaqMan^®^ MicroRNA Reverse Transcription kit (Applied Biosystems, USA) according to the manufacturer’s protocol.

Real-time PCR was performed with TaqMan^®^ MicroRNA assays (Applied Biosystems, USA) to analyze the expression of the following mature miRNAs: *miR-124*, *miR*-*132*, *miR*-*134*, and *miR*-*212*. The 15-μl reaction mixture contained 7.5 μl of 2× Universal PCR Master Mix, 0.75 μl of TaqMan MicroRNA assay, 1.75 μl H_2_O, and 5 μl of the RT product (diluted 1:15). Each reaction was run in duplicate in a 96-well plate with the following thermal conditions: 95 °C for 10 min, followed by 40 cycles of 15 s at 95 °C and 60 s at 60 °C. All PCR reactions were performed in a Bio-Rad CFX96 Real Time PCR Detection System, and the data were analyzed using CFX Manager Software 2.1. The relative amount of each miRNA was assessed using the comparative CT method (2^−ΔΔCt^) and normalization to the U6 small nuclear RNA (U6 snRNA). Because even subtle changes in the miRNA levels could have profound effects on their mRNA targets [32, 33], a fold-change cutoff of ≥1.3 (*p* < 0.05) was applied.

#### RT-qPCR for Gene Expression

A first-strand cDNA was generated using the total RNA (1 μg), hexamer primers and the Thermo Scientific Maxima First Strand cDNA Synthesis Kit in a final volume of 20 μl, according to the manufacturer’s recommendations.

The quantitative PCR assays were performed using the Thermo Scientific^™^ DyNAmo^™^ ColorFlash SYBRGreen qPCR Kit. The 10 μl PCR reaction mixture consisted of 5 μl of 2× master mix, 0.5 μl of the forward and reverse primers (Table 1), 2.5 μl of the RT product, and 1.5 μl of nuclease-free water. Duplicates of the standard samples and negative controls were prepared in a 96-well microplate and run in the Bio-Rad CFX96 Real-Time PCR Detection System. The following thermal profile was used: an initial activation step at 95 °C for 7 min, followed by 37 cycles consisting of a denaturation step at 95 °C for 15 s, primer annealing at 63 °C for 30 s, and amplification at 72 °C for 30 s. A melting curve analysis was performed after the qPCR was completed by collecting fluorescence data (65–95 °C for 5 s, with increments of 0.5 °C) for each primer.

**Table 1:** Primer sequences and the concentrations and sizes of the amplicon products from the real-time PCR reactions

Gene	Primers	Sequence (5′ → 3′)	Amplicon size (bp)
*Ago2*	Sense	GATATGCCTTCAAGCCTCCA	144
	Antisense	GAGGGCATTTCTCAGGTTTG	
*Pum2*	Sense	CTGGGATTTTCCTCCTCTCC	155
	Antisense	CCAGGTGCTGCAGAGATGTA	
*REST*	Sense	AATTTGAAGGCCAAACCCTT	132
	Antisense	GGCTTGCTTCTCTGCACTCT	
*Gapdh*	Sense	ATGACTCTACCCACGGCAAG	136
	Antisense	TACTCAGCACCAGCATCACC	

All primers were used at a concentration of 500 nM, except for the primers for *Ago2*, which were used at a concentration of 200 nM

To calculate the PCR efficiency of each pair of primers, five serial 10-fold dilutions of the cDNAs were prepared. The amplification efficiency (E) was determined from the linear slope of the dilution curve using the following equitation: E % = [10^(−1/slope)^ − 1] × 100 %. The ΔCt values for the target genes and reference gene (*GAPDH*) were calculated and the relative expression ratio was determined by the Pfaffl method [34]. The fold change was used to identify the differentially expressed genes; ≥1.3 and ≤ 0.6 were used as cutoff thresholds (*p* < 0.05).

#### Protein Extraction and Western Blot Analysis

The dissected rat dorsal striatum (10–20 mg) was disrupted and placed in RIPA buffer (Sigma, USA) containing 1 mM PMSF (Sigma, USA) and protein inhibitor cocktail (Sigma, USA). The homogenization step was performed for 2 × 90 s at 20 Hz using the TissueLyser II (Qiagen, USA), and then the samples were incubated for 30 min at 4 °C with gentle rotation. The lysates were centrifuged at 14,000×*g* for 30 min at 4 °C (to remove cellular debris), supernatants were collected, and the protein levels were measured with the BCA Protein Assay Macro Kit (Serva, Germany).

The expression of the Pum2, REST, Ago2, and GAPDH proteins was analyzed by immunofluorescence staining. Briefly, the proteins (100 ng) were mixed 1:1 with loading buffer (Fluka, Switzerland) and boiled at 95 °C for 5 min (Pum2 and Rest) or 60 °C for 15 min (Ago2). All samples were loaded on an SDS-PAGE gel (Fluka, Switzerland) and separated under constant voltage (90 V in the 3 % stacking gel; 120 V in the 10 % resolving gel) using the Mini-PROTEAN Tetra System (Bio-Rad, USA). Then, the REST and Pum2 proteins were transferred to PVDF membranes (Immobilon-FL Transfer Membrane, Millipore, USA) on the Criterion blotter (Bio-Rad, USA) at 100 V and constant current for 100 min. Alternatively, the Ago2 protein was transferred to the membrane using a semidry method with the following parameters: mixed range MW (25–150 kDa), three mini-gels, 9 min, 25 V, and 3.8 A. The *Pierce G2* Fast Blotter (Thermo Scientific, USA) was used for the semidry transfer. The membranes were blocked with Odyssey Blocking Buffer (LI-COR) diluted with 1× PBS (pH 7.5) for 1 h at room temperature and then incubated with specific primary antibodies overnight (with agitation) at 4 °C. The primary and secondary antibodies were diluted in the blocking buffer (prepared as described above) supplemented with 0.2 % Tween-20. The following primary antibodies were used at the indicated concentrations: 1:1000 of a rabbit monoclonal anti-Pum2 antibody (TA307097; OriGene, USA), 1:600 of a rabbit polyclonal anti-REST antibody (sc-25398; Santa Cruz Biotechnology, USA), 1:500 of a rabbit mAb against Argonaute 2 (C34C6) (Cell Signaling Technology, USA), and 1:1000 of a rabbit polyclonal anti-GAPDH antibody (sc-25778; Santa Cruz Biotechnology, USA). The blots were washed four times with PBST (1× PBST, 0.2 % Tween-20) prior to a 1 h incubation with the secondary antibody at room temperature (protected from light). With the exception of Ago2 detection, both the blocking and washing steps were performed using BSA and TBST (with 0.1 % Tween-20).

An infrared, fluorescently labeled secondary antibody (IRDye 680RD, goat anti-rabbit IgG, LI-COR; 1:5000) was used. The blots were washed and visualized at 700 nm with a LI-COR Odyssey^®^ Infrared Imaging System, and the results were analyzed using Image Studio v 2.1 software.

#### Statistical Analysis

All values are expressed as the means ± SEM. The behavioral data were analyzed by a two-way ANOVA for repeated measures followed by a *post hoc* Newman-Keuls’ test to determine the differences between the treatment conditions. The molecular data were analyzed using Student’s *t* test and one-way ANOVA with a *post hoc* Newman-Keuls’ test. The level of significance was set at *p* < 0.05. All statistical analyses were performed using GraphPad Prism version 5.04 software.

## Results

### Behavioral Analysis

After 14 daily cocaine self-administration sessions, the animals (groups labeled as SA-1 and SA-2; see Fig. 2 in the “Materials and Methods” section) exhibited a stable number of lever presses during the last 3 sessions, with a less than 10 % difference in their daily intake of cocaine. The mean number of cocaine infusions per day during the last 3 self-administrations days varied from 29 to 34; the animals received 186–222 mg/kg of cocaine throughout the experiment.

In the SA-1 group, the animals pressed the active lever more frequently, which was significantly different than the number of presses on the inactive lever, from the 5th through 14th experimental sessions (F_(13, 130)_ = 2.87, *p* < 0.001). In the last self-administration session, this group of rats displayed 191.5 ± 11.7 and 6.5 ± 3.1 active and inactive lever presses, respectively. In the SA-2 group, the rats pressed the active lever more frequently than the inactive lever from the fourth cocaine self-administration session through the third extinction day (F_(23,130)_ = 26.33, p < 0.001).

In the SA-2 group, the number of active and inactive lever presses during the last self-administration session and the 10th day of extinction training were 185.4 ± 10.8 and 2.0 ± 0.6, and 15.0 ± 2.0 and 7.4 ± 2.7, respectively. During the last 3 days of extinction training, the total number of active lever presses did not differ by more than 10 %.

In the YC-1, YC-2, YS-1, and YS-2 groups, the differences in the number of active versus the inactive lever presses failed to reach significance (data not shown). The YC-1 and YC-2 animals passively received exactly the same amount of cocaine (186–222 mg/kg) at the same time as the rats that had learned to actively inject cocaine (SA-1 and SA-2, respectively).

### Changes in the Expression Pattern of miRNAs

To assess whether cocaine induced long-lasting changes in the expression level of striatal miRNAs that are important for synaptic plasticity, we analyzed the expression pattern of *miR-124*, *miR-132*, *miR-134*, and *miR-212* after 14 days of the SA of the drug and after the 10-day extinction training (Table 2). The expression levels of *miR-124* and *miR-134* did not exceed the 1.3-fold cutoff threshold in the cocaine SA-1 rats and the YC-1 group compared to the YS-1 group or in the rats that underwent extinction training following cocaine SA-2 or YC-2 versus YS-2. Therefore, these miRNAs were excluded from the subsequent analyses.

**Table 2:** miRNA alterations in the rat striatum after cocaine self-administration and 10-day extinction training

miRNA	Group	Self-administration		Group	Extinction training	
		FC	*p* value		FC	p-value
*124*	**SA-1**	1.1 ± 0.05	0.225	**SA-2**	1.2 ± 0.07	0.020
*132*		**1.6 ± 0.12**	**0.001**		**1.7 ± 0.12**	**0.000**
*134*		1.2 ± 0.12	0.153		1.2 ± 0.03	0.001
*212*		**1.5 ± 0.12**	**0.002**		**1.5 ± 0.15**	**0.008**
*124*	**YC-1**	1.1 ± 0.14	0.362	**YC-2**	1.1 ± 0.04	0.201
*132*		1.3 ± 0.16	0.130		1.2 ± 0.05	0.004
*134*		1.0 ± 0.08	0.829		1.1 ± 0.05	0.200
*212*		1.0 ± 0.12	0.982		1.0 ± 0.08	0.864

The fold change (FC) values are reported as the means ± SEM (*n* = 6 animals per group); the *p* value was calculated using Student’s *t* test. miRNAs with FC ≥1.3 and *p* < 0.05 were considered to be differentially expressed (indicated in bold) compared to the YS group

*SA* self-administered group, *YC* yoked cocaine group, *YS* yoked saline (control) group

**Fig. 3:**
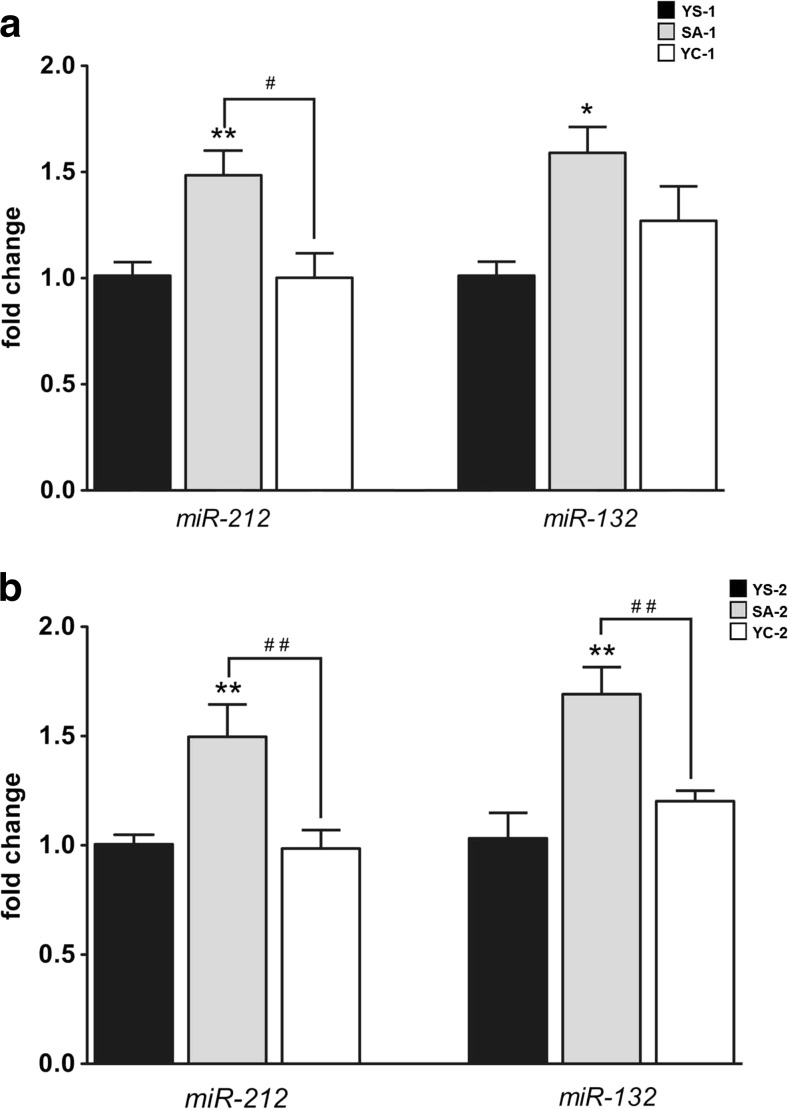
The expression pattern of *miR-212* and *miR-132* in the rat striatum after cocaine self-administration (**a)** and 10-day extinction training (**b**). *p < 0.05, **p < 0.01, compared to the yoked saline (control) group (YS); #*p* < 0.05, ##*p* < 0.01, comparisons between the self-administered (*SA*) and yoked cocaine (*YC*) groups (one-way ANOVA followed by the Newman-Keuls’ *post hoc* test; the data represent the means ± SEM, *n* = 6 animals/group)

We observed that the 14th day of cocaine SA had a significant effect on the level of *miR-212* (F_(2, 15)_ = 7.41, *p* < 0.01) and *miR-132* (F_(2, 15)_ = 5.50, *p* < 0.05). A *post hoc* analysis showed that active cocaine intake (SA-1), but not YC-1 administration, significantly increased the amount of *miR-212* (1.5-fold, *p* < 0.01) and *miR-132* (1.6-fold, *p* < 0.01) compared to YS-1 administration (Fig. 3a). Moreover, a statistically significant difference (*p* < 0.01) was observed in the level of *miR-212* between the cocaine SA-1 and YC-1 groups (Fig. 2a).

On the tenth day of extinction training, significant alterations in the level of *miR-212* (F_(2, 15)_ = 8.16, *p* < 0.01) and *miR-132* (F_(2, 15)_ = 11.26, *p* < 0.001) were also noted. Furthermore, the *post hoc* test indicated that the levels of *miR-212* and *miR-132* were only significantly upregulated in the rats with a cocaine SA history (1.5-fold, *p* < 0.01 and 1.7-fold *p* < 0.001, respectively) (Fig. 3b). Additionally, there were significant differences in the expression patterns of *miR-212* (*p* < 0.01) and *miR-132* (*p* < 0.001) between the cocaine SA-2 and YC-2 groups (Fig. 3b).

### Changes in the Gene Expression Levels

Real-time RT-qPCR was used to calculate the relative expression of the *Ago2*, *Pum2*, and *REST* genes after cocaine intake. The relative levels of the mRNA transcripts at two time points are shown in Table 3.

**Table 3:** The levels of the mRNA transcript in the rat striatum during cocaine self-administration and 10-day extinction training

miRNA	Group	Self-administration		Group	Extinction training	
		FC	*p* value		FC	*p* value
*Ago2*	**SA-1**	**0.6 ± 0.03**	**0.000**	**SA-2**	1.3 ± 0.05	0.000
*Pum2*		1.3 ± 0.03	0.000		1.2 ± 0.07	0.039
*REST*		**1.6 ± 0.08**	**0.000**		**1.4 ± 0.07**	**0.000**
*Ago2*	**YC-1**	**0.6 ± 0.07**	**0.000**	**YC-2**	1.3 ± 0.12	0.022
*Pum2*		1.0 ± 0.13	0.943		1.0 ± 0.08	0.747
*REST*		1.0 ± 0.13	0.741		1.2 ± 0.07	0.201

The fold change (FC) values are presented as the means ± SEM (*n* = 6 animals per group); the *p* value was calculated using Student’s *t* test. mRNA transcripts with FC ≥1.4 or ≤0.6 and *p* < 0.05 were considered to be differentially expressed (indicated in bold) compared to the YS group

*SA* self-administered group, *YC* yoked cocaine group, *YS* yoked saline (control) group

**Fig. 4:**
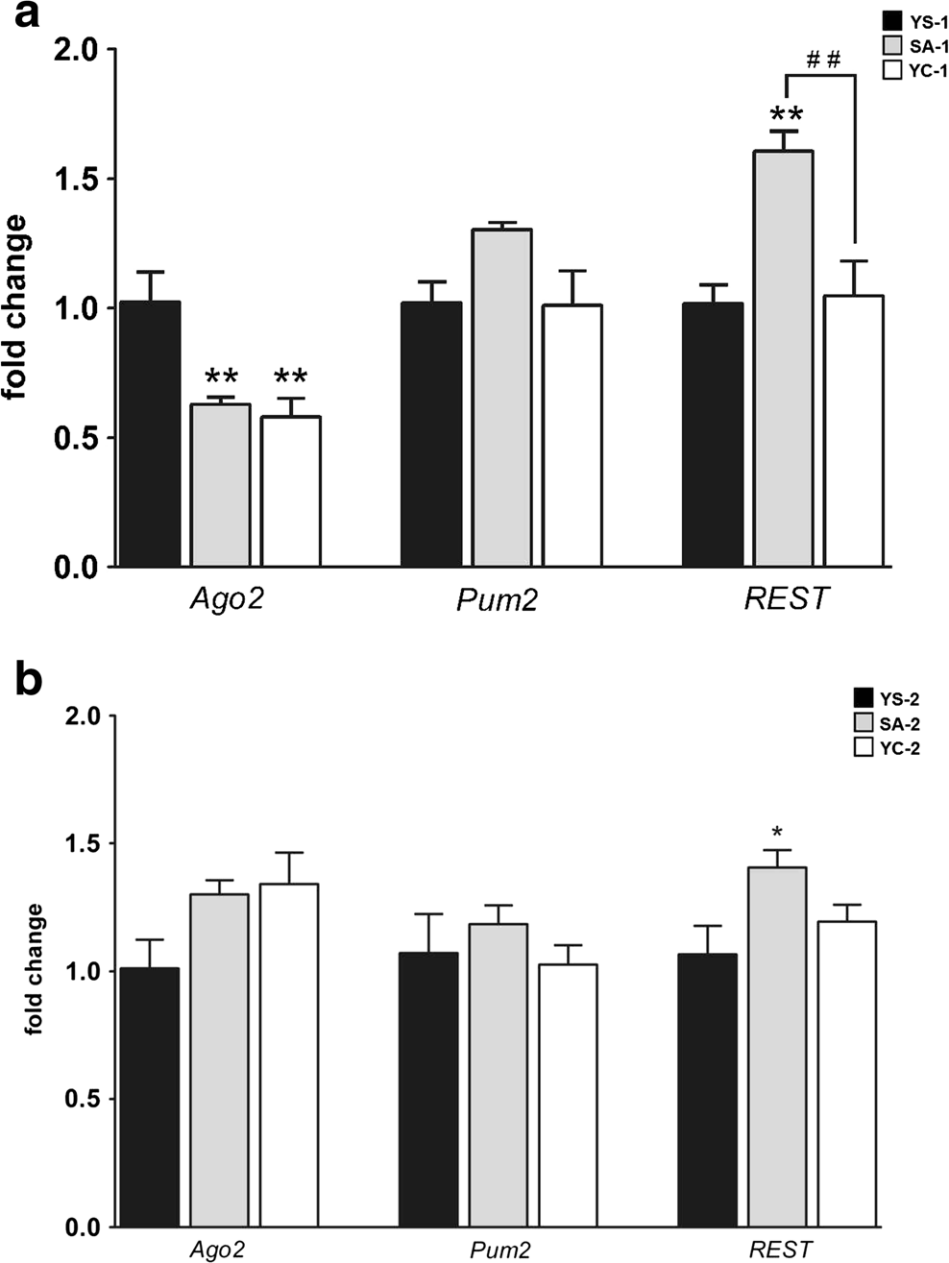
The relative amount of the mRNA transcripts in the rat striatum after cocaine self-administration (**a)** and 10-day extinction training (**b**). *p < 0.05, **p < 0.01, compared to the yoked saline (control) group (*YS*); ##*p* < 0.01, comparisons between the self-administered (*SA*) and yoked cocaine (*YC*) groups (one-way ANOVA followed by the Newman-Keuls’ *post hoc* test; the data represent the means ± SEM, *n* = 6 animals/group)

Cocaine SA and YC delivery induced a significant decrease in *Ago2* expression (F_(2, 15)_ = 9.05, *p* < 0.001) in the dorsal striatum in both cocaine-treated groups (SA-1 and YC-1) compared to the YS-1 control (Fig. 2a). In contrast, *Pum2* expression was not changed by cocaine (Fig. 4a). The level of the *REST* transcript was significantly changed (F_(2, 15)_ = 11.26, *p* = 0.001), and the subsequent *post hoc* analysis revealed a 56 % upregulation in the cocaine SA-1 compared to both the YC-1 and YS-1 groups (Fig. 4a).

After the 10-day extinction training, we did not observe significant changes in the *Ago2* and *Pum2* expression levels in the cocaine SA-2 and YC-2 groups compared to the YS-2 group (Fig. 4b). Interestingly, cocaine treatment altered *REST* expression in the rats (F_(2, 15)_ = 4.07, *p* < 0.05), and the *post hoc* test showed that the fold change in the cocaine SA-2 rats and their matched YS-2 counterparts reached significance (Fig. 4b).

### The Protein Expression Levels

**Fig. 5:**
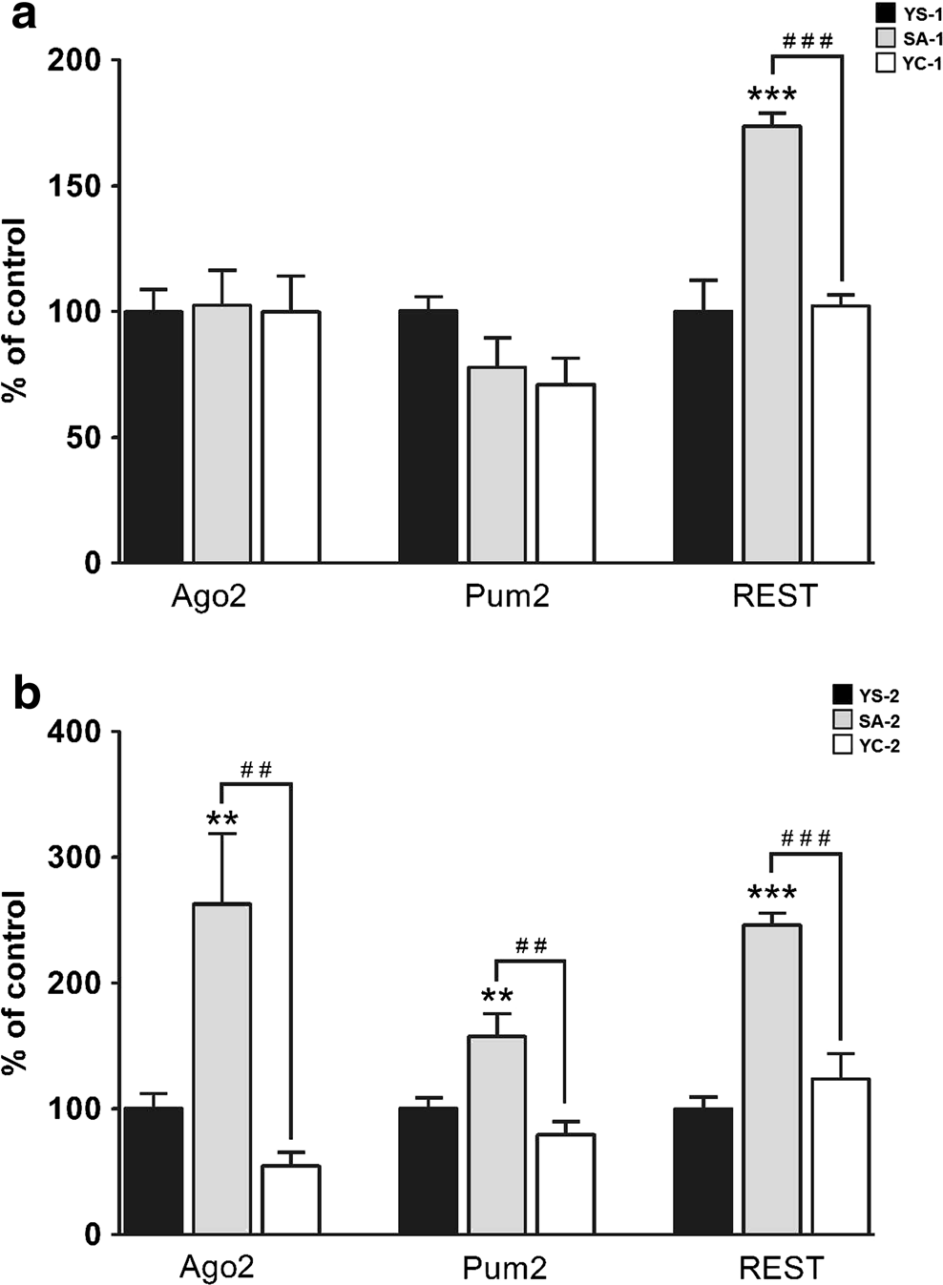
The results of the Western blot analysis in the rat striatum after cocaine self-administration (**a)** and 10-day extinction training (**b)**. **p < 0.01, ***p < 0.001, compared to the yoked saline (control) group (*YS*); ##*p* < 0.01, ###*p* < 0.01, comparisons between the self-administered (*SA*) and yoked cocaine (*YC*) groups (one-way ANOVA followed by the Newman-Keuls’ *post hoc* test; the data represent the means ± SEM, *n* = 5 animals/group)

To evaluate the possible changes at the protein level, we performed immunoblotting for the Ago2, Pum2, and REST proteins. After 14 cocaine SA sessions, only the levels of the REST protein were significantly changed (F_(2, 12)_ = 26.44, *p* < 0.001), the *post hoc* test further revealed significant differences between the cocaine SA-1 rats and their yoked controls (Fig. 5a).

Following the 10-day extinction training, we observed significant changes in the levels of the Ago2 (F_(2, 12)_ = 10.59, *p* < 0.01), Pum2 (F_(2, 12)_ = 9.76, *p* < 0.01), and REST (F_(2, 12)_ = 31.81, *p* < 0.001) proteins. Subsequently, the *post hoc* comparisons showed significant alterations in the cocaine SA-2 rats compared to both the YC-2 and YS-2 groups (Fig. 5b). It is worth noting that the levels of the REST protein and mRNA remained elevated not only following cocaine SA but also after the drug-free period during extinction training.

## Discussion

In the present study, we examined the levels of the miRNAs, mRNAs, and proteins that are essential for synaptic plasticity and/or learning adaptations in the dorsal striatum of rats subjected to cocaine SA and extinction training. The dorsal striatum was chosen because it serves as a key brain area for habit learning, the escalation of cocaine intake, and the compulsive drug-seeking behaviors [35].

Our results demonstrate the upregulation of mature *miR-132* and *miR-212* expression in the rats following cocaine SA, but not after 14 days of intake in either the YC or YS groups. The later finding extends the recent observations of Hollander et al. who demonstrated that 6-h (but not 1 h) access to cocaine SA for 7 days increased *miR-132* and *miR-212* expression in the rat striatum [19]. Surprisingly, the significant increase in the *miR-132* and *miR-212* levels was long-lasting and remained in the rats that had been withdrawn from cocaine SA. In fact, we report, for the first time, that the 10-day drug-free period during extinction training significantly enhances the expression of the studied miRNAs.

Possible explanations for the *miR-212 and miR-132* upregulation during the self-administration phase and its persistence during extinction training may include multiple cocaine-induced mechanisms, such as changes in dopamine and glutamate neurotransmission, alterations in specific signaling pathways, and/or epigenetic regulation. Using the same behavioral protocol with cocaine SA and extinction training, we previously demonstrated that cocaine SA significantly increased the extracellular accumbal DA levels compared to the YC controls [36]. Repeated cocaine SA also reduced the basal ventral striatal glutamate levels [36], which was linked with increased expression of the N2A subunit of the NMDA receptor in the rat striatum [37].

**Fig. 6:**
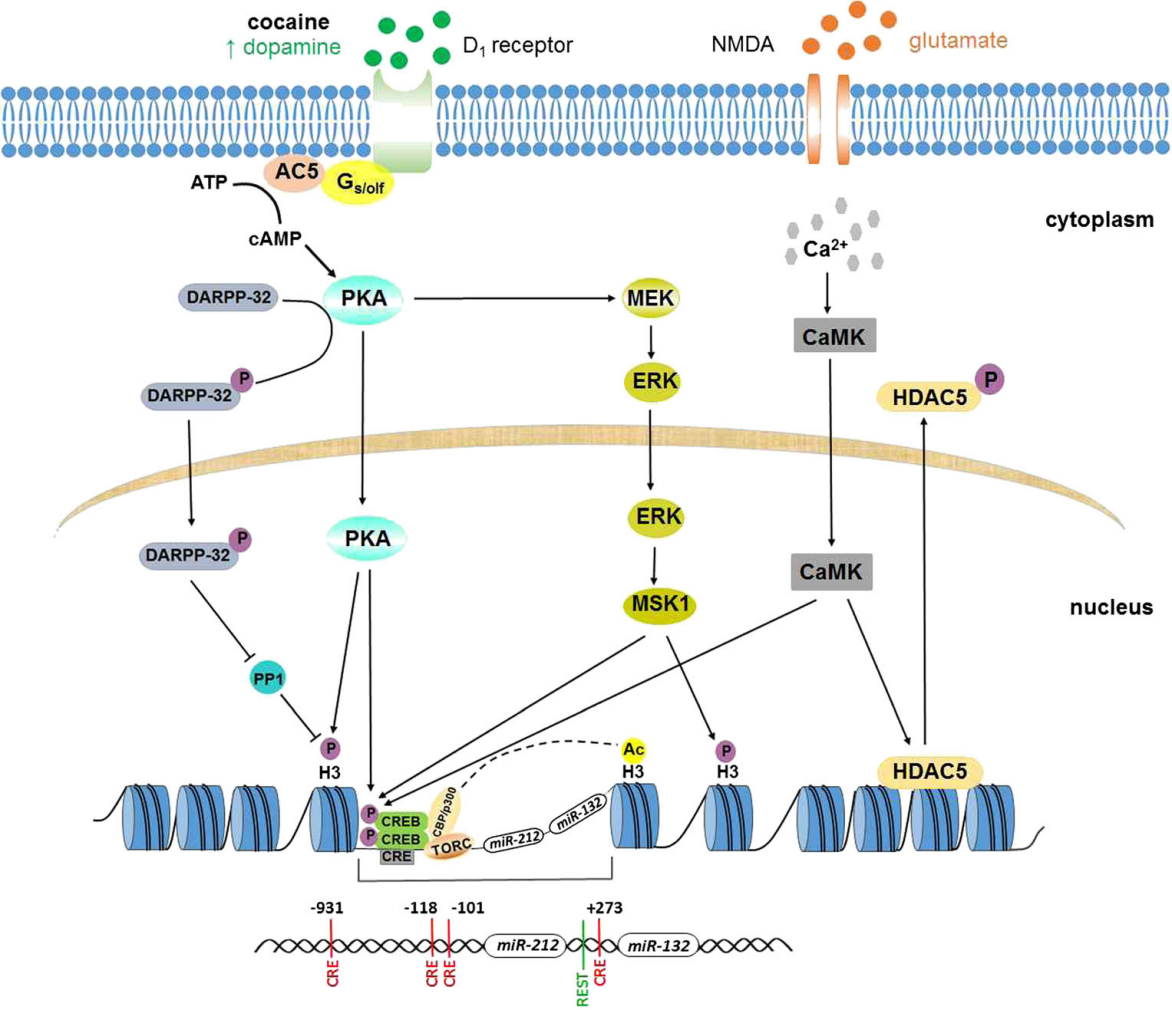
The putative signaling pathways involved in long-term *miR-212/132* expression. Cocaine increases the dopamine levels in the synaptic cleft. D1 receptor stimulation increases the intracellular cAMP levels, which activates protein kinase A (PKA). PKA can directly or indirectly phosphorylate CREB and the histone tails via the ERK-dependent MSK1 signaling cascade [38, 39]. NMDA receptor stimulation by glutamate promotes the influx of Ca^2+^ that is necessary for the activation of the CaMK kinase cascade that influences CREB and HDAC phosphorylation. CREB binds to the CRE sites as a dimer; its phosphorylation (at Ser-133) allows it to interact with coactivators, such as CBP/p300 and CRTCs (TORCs) [40]. The CBP/p300 protein family possesses histone acetyltransferase activity [41], interacts with large transcriptional complex and regulates transcription via chromatin remodeling that enables transcription factors to access the DNA template. [40]. TORCs are essential coactivators; however, they do not influence CREB phosphorylation, but are required for active CRE-dependent expression [42]. Moreover, MSK1 kinase expression is enriched in the striatum [43] and seems to play an important role in histone H3 and CREB phosphorylation [38]. DARPP-32, which is phosphorylated by PKA, accumulates in the nucleus of striatal neurons and inhibits PP1, leading to increased histone H3 phosphorylation [44]

As chronic cocaine exposure alters dopamine and glutamate signaling by stimulating the dopamine D1 and glutamate NMDA receptors, these targets trigger the activation of the corresponding downstream signaling pathways and lead to CREB-dependent gene expression (Fig. 6). Interestingly, four different CRE sites and one REST site are present within the regulatory region of the *miR-212/132* cluster (Fig. 6). A recent study has elegantly demonstrated that *miR-212* is highly responsive to increased expression of CREB and its essential coactivators TORCs and Ser-133 phosphorylation in the dorsal striatum 24 h after the last cocaine self-administration session in rats with extended access to the drug [19]. Furthermore, *miR-212* may amplify CREB activity through positive feedback signaling; *miR-212* activates Raf1 (the kinase that phosphorylates and sensitizes adenylyl cyclase), at least in part, via SPRED1 repression (a Raf1 suppressor) [19]. Finally, the Raf1-mediated increased expression of TORC may enhance CREB/TORC signaling. The hypothesis that *miR-212* may regulate its own (long-lasting) expression during cocaine SA by enhancing the activity of the CREB/TORC signaling cascade seems promising; however, it needs to be tested.

The observed upregulation of *miR-212* and *miR-132* in the dorsal striatum following extinction training in rats with a history of cocaine SA may be associated with posttranslational modifications (PTMs) of histones that are induced by daily cocaine administration and the subsequent changes in dopamine and glutamate transmission (see Fig. 6). In fact, there are several studies showing that cocaine alters the expression level of some histone-modifying enzymes within key brain reward regions, including the striatum, and the changes in the chromatin structure may persist for extended periods after cocaine withdrawal [45–47]. Thus, chronic cocaine administration may activate histone acetyltransferases (HATs) or inhibit histone deacetylases (HDACs) [48]. Kumar et al. demonstrated that chronic cocaine SA increased the level of histone H3 acetylation (at the *Bdnf* and *Cdk5* gene promoters) in the rat striatum, which persisted 1 week after cocaine withdrawal [45]. Interestingly, the level of *Bdnf* promoter acetylation was 4-fold higher after 1 week of withdrawal compared to 1 day of drug withdrawal. Additionally, chronic cocaine (experimenter administration or SA) triggered HDAC5 phosphorylation in the ventral striatal neurons, causing it to shuttle out [46, 49, 50], and the hyperacetylation of histones at certain genes persisted for weeks after the last drug exposure [48]. Recent studies also indicated histone phosphorylation and methylation following repeated cocaine exposure [47, 50, 51]. Cocaine-enhanced phosphorylation of histone H3 at serine 10 (H3S10) in the mouse striatum was mediated by the D1 receptor and the subsequent direct MSK1 signaling pathway [38], as well as by nuclear shuttling of DARPP-32 in striatal neurons [44]. Following repeated cocaine SA, two histone methyltransferases, G9a (Ehmt2) and GLP (Ehmt1), were downregulated in the mouse ventral striatum [52]. As a result, monomethylation and dimethylation of lysine 9 on histone H3 (H3K9me1 and H3K9me2) were also reduced. A recent study has revealed that G9a expression was significantly decreased in both Drd1 and Drd2 mouse GABAergic striatal neurons in response to repeated cocaine treatment and remained reduced for several days during the drug withdrawal period [53]. Consistent with the above cited findings, it is very likely that the long-term upregulation of *miR-212* and *miR-132* is governed by epigenetic mechanisms.

**Fig. 7:**
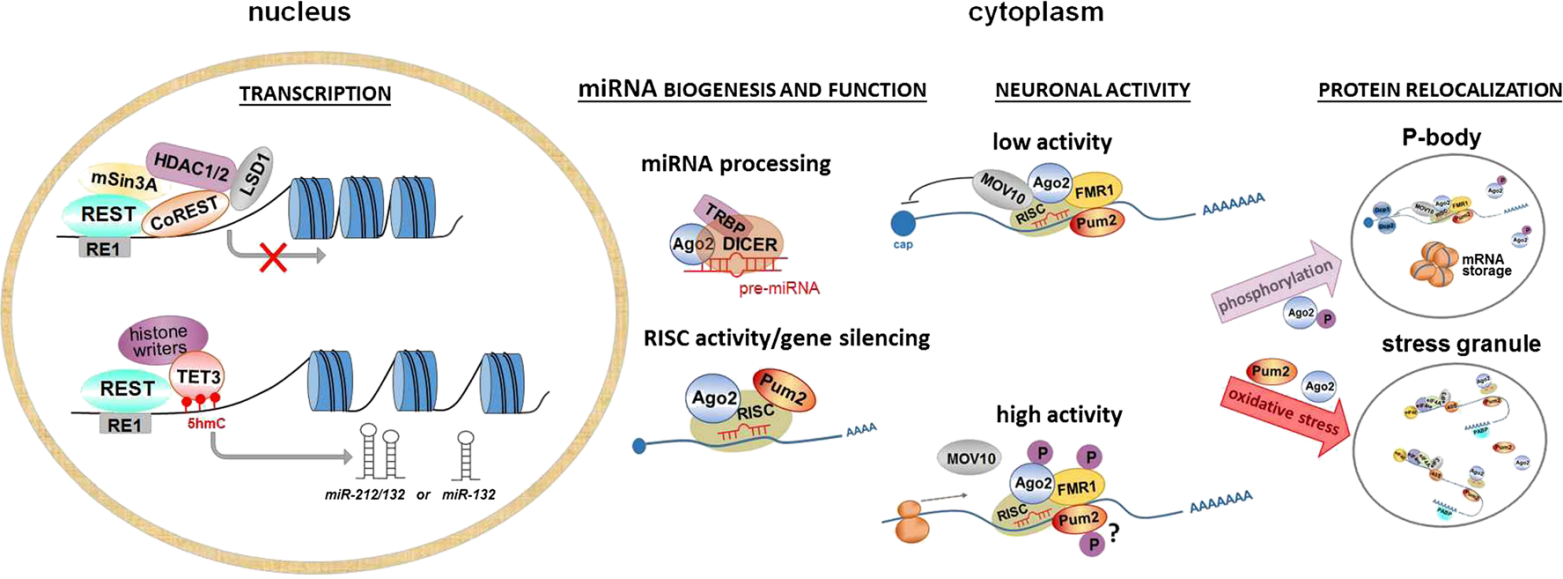
A putative mechanism for the interaction between miRNA and the Ago2, Pum2, and REST proteins. *Transcription*: *(i)* REST interacts with mSin3A and CoREST, which, in turn, recruits the HDAC complex (HDAC1 and HDAC2). Additionally, CoREST recruits LSD1. The HDACs deacetylate and LSD1 demethylates the lysine residues of the nucleosomal histone core; these events condense the chromatin and limit the accessibility to the DNA, resulting in the inhibition of translation (indicated schematically by an X) [54]. *(ii)* REST recruits TET3 and stimulates its hydroxylase activity to form 5hmC. Subsequently, TET3 interacts with histone writers (e.g., NSD3, NSD2, and SETD2) and mediates histone H3 methylation [55]. This mechanism may be involved in the expression of either *miR-212/132* cluster or *miR-132* alone*. miRNA biogenesis and function*: *(i)* Ago2 interacts with Dicer and TRBP to efficiently catalyze pre-miRNA cleavage; *(ii)* Pum2 mediates the activity of the RISC complex in neurons [56], and, together with RISC and miRNAs, Ago2 participates in gene silencing. *Neuronal activity*: *(i)* Under low-activity conditions, Ago2 and its associated RNA-binding proteins (FMR1 and Pum2) suppress protein production by blocking cap-dependent translation. *(ii)* Following synaptic stimulation, posttranslational modifications of Ago2, FRM1, and Pum2 (?) are involved in the remodeling of the miRISC complex, leading to efficient mRNA translation [56]. *Protein relocalization*: *(i)* P-bodies may regulate local transcription in the synapses by managing miRNA-mediated regulation of mRNA transcription. In the P-bodies, the mRNAs are targeted for storage and/or degradation. Upon synapse activation, a portion of the mRNAs stored in the dendritic P-bodies may reenter the translational pathway [57]. Phosphorylation of the Ago2 protein (at Ser-387) facilitates its localization to the P-body [58]. *(ii)* Stress granules (SGs) are generated in response to stressful environmental conditions and are another reservoir of mRNAs. SGs contain mRNAs, translation initiation factors (e.g., eIF3, eIF4A, and eIF4G), small ribosomal subunits and poly(A) binding protein (PABP) proteins as well as the mRNA decay machinery. OS represses the translation of important neuronal genes and influences Ago2 and Pum2 accumulation in the SGs

Our next important findings include the changes in the levels of the Ago2, Pum2, and REST mRNAs and proteins following cocaine treatment. These proteins are important for controlling dendritic spine morphology and synaptic plasticity, and they regulate the expression, processing, and function of many miRNAs, such as *miR-212/132* (Fig. **7**). We observed a significant decrease in the levels of the *Ago2* transcript in the striatum (∼40 %) during cocaine SA and in the YC group; however, this did not correlate with Ago2 protein expression. Interestingly, extinction training resulted in a 2-fold increase in the Ago2 protein level in the rats that self-administered cocaine, while a non-significant elevation in the levels of the *Ago2* mRNA were observed in rats that had previously experienced cocaine. The observed inconsistency in the Ago2 transcript and protein levels may be linked to posttranslational modifications of Ago2 that affect its stability and activity. Indeed, Ago2 is modified by hydroxylation, phosphorylation, and ubiquitination. Hydroxylation of Ago2 at proline-700 has an impact on its stability and subcellular localization, as well as on effective siRNA-mediated RNAi [59]. Similarly, p38/MAPK-mediated phosphorylation of Ago2 at serine-387 facilitates its localization to processing bodies [58]. Oxidative stress caused by reactive oxygen species (ROS) may activate the MAPK pathways [60], and we show that the level of superoxide dismutase activity was significantly enhanced in the dorsal striatum following cocaine SA, but not in the YC rats [61]; therefore, the latter finding suggests that cocaine SA may evoke more potent oxidative damage due to the increased release of dopamine [62] than passive cocaine administration. Interestingly, recent in vitro studies demonstrated that translation inhibition did not reduce the level of the Ago2 protein within 48 h after the inhibitor treatment [59], and the protein half-life was greater than 5 days [63]. Detzer at al. also showed that cellular stress (oxidative or translational) is related to the accumulation of human Ago2 in stress granules [59]. Taken together, we assumed that the cocaine SA-induced ROS production may activate MAPK signaling, resulting in Ago2 phosphorylation, which influences its stability and relocalization during extinction training. Further studies are necessary to verify our above hypothesis and to confirm the relevance of Ago2 in the regulation of miRNA and mRNA after cocaine SA and withdrawal.

Currently, there are limited data in the literature that evaluate the impact of cocaine on the *Ago2* mRNA and protein levels; moreover, all of these studies analyzed mice. Thus, Ago2 deficiency in mouse Drd-2-expressing neurons significantly decreased the daily cocaine SA by reducing the animal’s motivation [22]. In accord with this finding, chronic passive cocaine treatment in mice significantly increased the expression levels of *Ago2* mRNA in the NAc and Ago2 protein in the striatal PSD-enriched fraction, which paralleled the alterations in the levels of specific miRNAs [23]. Based on these observations, it is very likely that cocaine—by increasing the level of synaptically localized Ago2 protein and miRNAs—influenced stability and/or the translation rate of mRNA at synapse. The previous reports seem to be in contrast to our present findings; however, these discrepancies may be linked with cellular fractionation procedures (PSD fraction vs. whole lysate) and/or with selective deficiency in Ago2 in the population of Drd-2-expressing neurons. The used conventional knockout approach is associated with changes in brain development and maturation, and therefore, is unknown if Ago2 deficiency before cocaine exposure altered functional responses in the firing of the D_2_ receptor-positive medium spiny neurons within ventral striatal region that in wild-type animals suppresses cocaine reward by mediating punishment [64, 65]. Based on these observations, we concluded that the increased levels of Ago2 protein during extinction training in the rats with history of cocaine SA cannot be considered as a marker of cocaine intake (lack of changes in YC rats), but instead is linked to the motivation for or context of cocaine administration. In the context of our results, it should be underlined that extinction training is a new form of learning that generates a new memory linked with extensive synaptic reorganization and neuroadaptations by modulation gene and protein expression [66]. Whether increased Ago2 levels are the trigger of drug relapse or adaptive protein to environmental changes in animal cells is not yet known.

Our present data also indicated an increased level of the Pum2 protein during extinction training in the rats with history of cocaine SA, although the transcript level was similar to the YS control. The in vivo function of Pum2 is still poorly understood. A separate study demonstrated that Pum2 plays a key role in neuronal excitability and the behavioral responses to environmental changes [67]. Schratt highlighted that synaptic stimulation influences Pum2 activity during pre-miRNA processing and suppresses protein synthesis [27]. Because Pum2 represses the translation of important neuronal mRNAs, contributes to the miRNA pathway [27], and is contained in dendritic stress granules [26], it is tempting to speculate that its increased levels following extinction training may result from alterations in neuronal activity and may be crucial for the spatial and temporal expression of proteins that are required for synaptic plasticity.

For the first time, we also reported that both the REST transcript and protein levels were significantly increased during cocaine SA and extinction training, while in the YC rats, they reached the YS control levels. We propose that the increase in the REST levels in cocaine SA could be related to the motivational aspect of drug delivery and/or the association of drug-related stimuli. REST is known as a transcriptional repressor, which controls thousands of target genes involved in neuronal development and function and is important not only in the embryo but also in adult neurons.

Comparative sequence analysis revealed 895 candidate RE1 sites in human genome [68] and in silico screen of *Xenopus tropicalis* genome identified 742 RE1 motifs; some of them are common in human and mouse [69]. The target REST genes important for neuron functions include those that encode ion channels [70], neurotransmitter receptors (e.g., subunits of the NMDA and AMPA receptors) [71, 72], neurotrophins and their receptors [73], synaptic vesicle proteins [74], and cytoskeletal and adhesion molecules [75]. REST mediates transcriptional repression via chromatin remodeling; it operates as a scaffold to assemble and position several cofactors, including mSin3A, histone deacetylase (HDAC1/2), and demethylase (LSD1) and its corepressor CoREST [55, 76, 77]. On the other hand, emerging evidence reveals that REST may act also as an transcriptional activator via interaction with TET3 [78] or double-stranded ncRNAs [75]. In addition, the REST levels also control the expression of splicing factors; therefore, it is indirectly involved in the formation of alternatively spliced isoforms, which can attenuate its repressive ability [55, 79]. The REST protein influences the expression of splicing factors (e.g., *mSR100* and *NOVO*) and alters the structure and function of synapses [39]. Another interesting finding suggested that glutamate-induced oxidative stress [73, 74] and prolonged in vitro depolarization of primary neuronal cultures upregulate REST expression. Taken together, these results indicate that REST plays crucial and complex roles in the regulation of the expression of neuronal genes that are involved in synaptic plasticity. We strongly believe that REST protein through its targets and coordinated feedbacks with brain-related miRNAs can be an important element of molecular basis of cocaine use disorder; however, further analysis with using knockdown or overexpression approaches is requested to confirm it.

### Concluding Remarks

For the first time, our data showed that cocaine SA and extinction training is able to significantly increase the expression levels of the mature *miR-212* and *miR-132* transcripts in rats.

Furthermore, the elevated level of the *REST* mRNA that was only observed during cocaine SA may result from motivational and not pharmacological effects of cocaine and may be regulated at the epigenetic level. We postulate that the long-term upregulation of the *miR-212/132* and *REST* mRNA levels is due to chromatin remodeling; however, additional studies, including chromatin immunoprecipitations, should be performed to determine the changes in histone modifications close to these loci.

Our findings also showed that the levels of the Ago2 and Pum2 proteins (but not mRNAs) were significantly increased during extinction training in rats with a history of cocaine SA. The increased protein levels may be due to the molecular changes associated with new learning, memory formation, posttranslational modifications, which may influence protein stability and subcellular localization and oxidative stress. We realize that our study does not deliver direct evidence for relationship between increased level of REST, Ago2, selected miRNAs, and addictive behavior. However, these results suggest that REST could be a new player essential for cocaine use disorder.
